# Oxidation and Reduction of Polycrystalline Cerium Oxide Thin Films in Hydrogen

**DOI:** 10.1021/acs.jpclett.3c01662

**Published:** 2023-08-10

**Authors:** Adva Ben Yaacov, Lorenz J. Falling, Roey Ben David, Smadar Attia, Miguel A. Andrés, Slavomír Nemšák, Baran Eren

**Affiliations:** † Department of Chemical and Biological Physics, Weizmann Institute of Science, 234 Herzl Street, 76100 Rehovot, Israel; ‡ Advanced Light Source, Lawrence Berkeley National Laboratory, Berkeley, California 94720, United States; § Materials Science Division, Lawrence Berkeley National Laboratory, Berkeley, California 94720, United States; ∥ Nuclear Research Centre—Negev, Beer-Sheva 84190, Israel; ⊥ Department of Physics and Astronomy, University of California, Davis, California 95616, United States

## Abstract

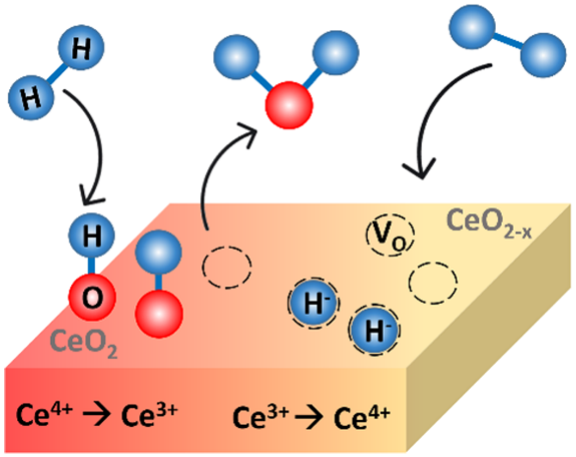

This study investigates the oxidation state of ceria thin films’ surface and subsurface under 100 mTorr hydrogen using ambient pressure X-ray photoelectron spectroscopy. We examine the influence of the initial oxidation state and sample temperature (25–450 °C) on the interaction with hydrogen. Our findings reveal that the oxidation state during hydrogen interaction involves a complex interplay between oxidizing hydride formation, reducing thermal reduction, and reducing formation of hydroxyls followed by water desorption. In all studied conditions, the subsurface exhibits a higher degree of oxidation compared to the surface, with a more subtle difference for the reduced sample. The reduced samples are significantly hydroxylated and covered with molecular water at 25 °C. We also investigate the impact of water vapor impurities in hydrogen. We find that although 1 × 10^–6^ Torr water vapor oxidizes ceria, it is probably not the primary driver behind the oxidation of reduced ceria in the presence of hydrogen.

The interaction of H_2_ with oxide surfaces is a fundamental process of the utmost importance. Among the various consequences of this interaction, H_2_-splitting is particularly significant, which can occur through different mechanisms, depending on the conditions and the nature of the oxide. These include homolytic hydride mechanism (eq 1), homolytic hydroxyl mechanism (eq 3), and a heterolytic mechanism (eq 5). Additionally, surface hydroxyls can be categorized as bridging hydroxyls or terminal hydroxyls, the former type typically leaving an oxygen vacancy (V_O_) on the surface upon desorption (eq 4). Hydride formation can be reversed by annealing in vacuum (eq 2). The following equations summarize the H_2_-splitting and gas desorption mechanisms on oxidized and reduced ceria:

Homolytic hydride formation
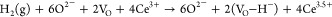
1

Recombinative H_2_ desorption (reverse of eq 1)
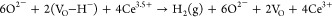
2

Homolytic hydroxyl formation
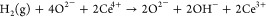
3

Oxygen vacancy formation via recombinative H_2_O desorption
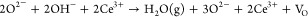
4

Heterolytic hydride and hydroxyl formation
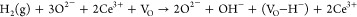
5

H_2_-splitting on ceria surfaces has garnered considerable attention in recent years due to its relevance in catalytic hydrogenation reactions. In a series of *ex situ* X-ray photoelectron spectroscopy (XPS) studies, the chemical state of ceria during the interaction with H_2_ was assigned via the valency of the Ce^*n*+^ cation.^1,2^ The presence of various H-containing species was detected by *in situ* neutron scattering and nuclear reaction analysis (NRA) that are both powerful techniques for detecting hydrogen but are not surface-sensitive.^3,4^ Interestingly, *in situ* infrared (IR) spectroscopy measurements on highly ordered ceria thin films have not detected the IR-active Ce–H stretching band, but this band has been recently detected for ceria powders.^1,2^ Recent works suggest that the type of hydrogen species may influence the activity and selectivity of hydrogenation reactions.^5^

Seminal studies have utilized stoichiometric CeO_2_(111) and reduced CeO_2-*x*_(111) thin films grown on Ru(0001) as model systems.^1,2,4^ In the first study, NRA performed after exposing the sample to 7.5 Torr H_2_ demonstrated that H_2_ dissociation takes place at the surface of CeO_2_(111) to form H-containing hydroxyl species only on the surface, whereas other H-containing species are formed in the subsurface of CeO_2-*x*_(111).^4^ Further studies were performed with a combination of *in situ* IR reflection absorption spectroscopy (IRRAS) and *ex situ* XPS in vacuum after exposing ceria to 7.5 Torr H_2_ (or D_2_).^1,2^ H_2_ was found not to interact with CeO_2_(111) at 27 °C,^2^ whereas on CeO_2-x_(111) formation of hydrides (eq 1) was inferred from the change in the oxidation state of ceria.^1,2^ Upon heating the sample to 177 °C in vacuum, this oxidation process on CeO_2-x_(111) is partially reversed (eq 2).^1^ At 127 °C in H_2_, small amounts of hydroxyls and reduced metal centers are formed on the stoichiometric CeO_2_(111) surface (eq 3), where the hydroxyls desorb once H_2_ is removed (eq 4).^2^ The reduced CeO_2-*x*_(111) surface, on the other hand, becomes oxidized at 127 °C due to the formation of hydride species as discussed above for 27 °C.^2^ However, this oxidation process is less prominent at 127 °C than 27 °C because of the competing hydroxyl formation that reduces the surface, which is not significant at 27 °C.^1^ At temperatures of 227 °C and above, both CeO_2_(111) and CeO_2-*x*_(111) surfaces exhibit a high concentration of Ce^3+^ ions, resulting from desorption of the surface hydroxyls and/or the hydride species diffusing into the bulk.^2^ In other words, at 227 °C and above V_O_ formation happens on CeO_2_(111) via recombinative desorption of surface hydroxyls, which were formed by H_2_ dissociation (eqs 3 and 4), and thus there is no longer any significant difference between CeO_2_(111) and CeO_2-*x*_(111).^2^

Interaction of H_2_ with ceria was also studied using model powder catalysts. At 27 °C, reduced surfaces of ceria powders were found to behave similar to the CeO_2-*x*_(111) surfaces,^1^ with the addition of the kinetically favored heterolytic dissociation (eq 5).^2^ At 200 °C, it was found that the formation of Ce^3+^ via desorption of H_2_O molecules (eqs 3 and 4) is counteracted by a further reaction of hydride formation (eq 1) and thereby oxidation to Ce^4+^ species.^2^ At temperatures exceeding 500 °C, the chemistry becomes more complex with the presence of bulk hydroxyl and hydride formation detected using electron spin resonance (ESR) and IR spectroscopies, respectively.^2^ Furthermore, combined thermal gravimetric analysis (TGA) and thermal desorption spectroscopy (TDS) studies suggest the rate-limiting step of ceria reduction at such high temperatures to be the formation of H_2_O.^6^ Bulk hydroxyls in the presence of H_2_ were also reported at 800 °C.^7^ In practice, whether one or more of the H_2_-splitting mechanisms take place depends on the H_2_ pressure, temperature, and the density of V_O_.^6^

Previous XPS studies investigating the H_2_-ceria interaction have largely been conducted *ex situ*,^1,2^ which may introduce inaccuracies due to changes in surface chemistry during sample transfer in vacuum. Here, we prepared a polycrystalline CeO_*x*_ thin film and studied its interaction with H_2_ by using core-level Ce 4d spectra and valence-band spectra. Both show oxidation in the presence of H_2_ (25–350 °C) and reduction while annealing in vacuum (up to 450 °C). At elevated temperatures (400–450 °C) in the presence of H_2_, both these processes and reduction through hydroxyl formation take place simultaneously. We also considered the potential influence of H_2_O impurities in the gas mixtures, as they can affect the surface chemistry.^8,9^ Oxidation through hydride formation and through adsorption of H_2_O vapor impurities show different temperature-dependent trends, and their effects are different at different depths of the sample.

A 25 nm thick polycrystalline ceria thin film prepared by e-beam evaporation on a highly doped silicon wafer piece was used as our sample. The substrate was kept at 25 °C during deposition. The deposition rate was 0.2–0.3 Å/s. AP-XPS measurements were performed at the beamline 9.3.2 of the Advanced Light Source (ALS) in Berkeley, which offers a lower flux density (5 × 10^7^–2 × 10^8^ photons/cm^2^/s) compared to most other AP-XPS beamlines, and therefore has a lower risk of beam-induced effects.^10^ Photon energies (*E*_*h*υ_) were chosen as 380, 530, and 780 eV, respectively, for the Ce 4d, C 1s, and O 1s core-level regions, to obtain a photoelectron kinetic energy (*E*_kin_) of roughly 250 eV for each element. Ce 4d region was also measured with *E*_*h*υ_ = 780 eV to obtain the oxidation state of cerium with increased subsurface sensitivity. Valence band spectra were acquired at *E*_*h*υ_ = 380 eV and *E*_*h*υ_ = 780 eV. All spectra were acquired using a Scienta HIPP hemispherical analyzer with a pass energy of 100 eV. All the collected raw data are shown in Figures S1–S7 of the Supporting Information after shifting their positions using reference peak positions. The measurement chamber was prepared with a conventional bake-out, and the base pressure was 1 × 10^–9^ Torr prior to any gas dosing. However, the base pressure increased after each experiment due to the presence of residual H_2_O vapor. The typical base pressure during the experiments ranged between 3 × 10^–9^ and 1 × 10^–8^ Torr.

Prior to AP-XPS experiments, the samples were outgassed at 200 °C in the preparation chamber. The samples were then oxidized by heating in an O_2_ atmosphere and reduced by thermal annealing in vacuum. H_2_ was dosed onto both the oxidized and reduced samples. Gases such as H_2_, O_2_, and H_2_O vapor were dosed through leak valves. Milli-Q H_2_O was prepared as liquid via freeze–pump–thaw cycles to remove dissolved gases. 99.9999% purity H_2_ and O_2_ gases were used, and the gas lines were cleaned via several cycles of pumping and purging prior to their use.

The Ce 4d core-level lines were primarily utilized to determine the changing oxidation state of ceria in the presence of H_2_ at various temperatures. This enabled us to draw conclusions about the reactions presented in eqs 1–4, however we cannot draw any conclusion regarding the reaction in eq 5 because the valency of the Ce^*n*+^ cations does not change with this reaction. Details regarding the O 1s core-level lines can be found in the Supporting Information. Figures 1a and 1b illustrate examples of the Ce 4d spectra acquired in this work at different conditions using *E*_*h*υ_ = 380 and 780 eV. For the Ce 4d region, these photon energies result in an inelastic mean free path (IMFP) of electrons in CeO_2_ of approximately 0.7 and 1.2 nm, respectively.^11,12^ Voigt lineshapes were employed after Shirley background subtraction to fit the spectral region using eight peaks. The full width at half-maximum (fwhm) and relative positions of all the peaks were constrained to be consistent between the Ce 4d spectra acquired at different conditions. The purple peak in Figures 1a and 1b is due to Si 2p peak of the native oxide of the silicon wafer substrate, which remains unaffected by the changing conditions. The normalized intensity of the Si 2p peak is around one-third of the normalized total intensity of the Ce 4d peaks. Such a high Si 2p intensity is because of the relatively low packing factor of the ceria thin films (Figure S10), and therefore, some of the underlying Si is exposed. Similar to the approach in ref (13)., we fixed the position of the Si 2p peak to 102.5 eV and assigned the peaks related to ceria species accordingly. The red peaks that are slightly above 123 and 126 eV represent the states characteristic for the Ce^4+^ cations with a 3.3 eV spin–orbit splitting.^13^ The blue feature, slightly above 105 eV and of low intensity, is absent under oxidizing conditions. This feature serves as a characteristic feature of the Ce^3+^ cations, as it does not overlap with any of the Ce^4+^ related features.^14^ The remaining features in Figures 1a and 1b lie between the aforementioned peaks and are depicted as four yellow peaks. They have contributions from both the Ce^4+^ and Ce^3+^ cations, and their origin is the subject of dispute in the literature due to the strong coupling between the Ce 4d and Ce 4f levels, resulting in multiplet splitting.^13,14^ The relative intensities of the yellow peaks among each other differ for the Ce^4+^ and Ce^3+^ chemical states; however, we did not utilize these peaks in further analyses to avoid ambiguities. It should also be noted that the analysis of the blue feature at 105 eV is less reliable than the others due to its low intensity.

**Figure 1:**
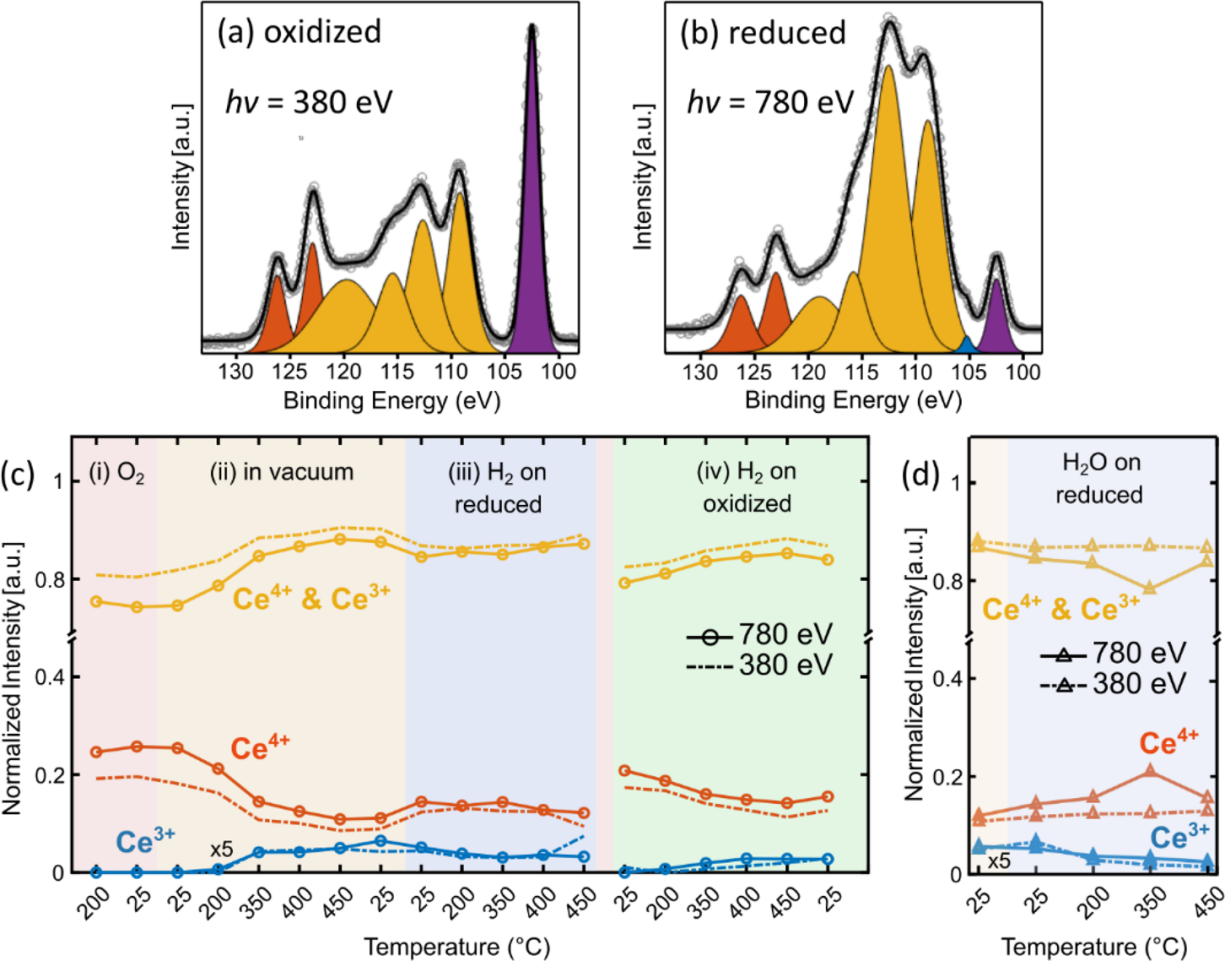
(a, b) Examples of data fitting of the Ce 4d features after background subtraction. (a) Acquired with *E*_*h*υ_ = 380 eV when the sample was at 200 °C in the presence of 0.01 Torr O_2_, whereas (b) Acquired with *E*_*h*υ_ = 780 eV when the sample was at 450 °C in the presence of 0.1 Torr H_2_. The purple peak is due to SiO_2_. Red features are due to the Ce^4+^ chemical state, and the weak blue feature is due to the Ce^3+^ chemical state. Yellow features are disputed in the literature, and they are assumed to have contributions from both Ce^4+^ and Ce^3+^ chemical states in the present study. The intensity ratios of the Si 2p and Ce 4d features are different in parts a and b due to different photoionization cross sections (σ) at different photon energies (i.e., σ_Ce 4d_/σ_CeSi 2p_ is ∼1.5 @380 eV and ∼4.5 @780 eV). In parts a and b, the open circles are the data points, whereas the solid black line is the sum of all fitted peaks. (c) The normalized intensity of the Ce 4d features measured at both *E*_*h*υ_ = 380 eV and *E*_*h*υ_ = 780 eV as a function of condition. (d) The normalized intensity of the Ce 4d features measured at both *E*_*h*υ_ = 380 eV and *E*_*h*υ_ = 780 eV during the test experiment with 10^–6^ Torr H_2_O vapor dosed onto a thermally reduced ceria surface. On reduced samples, in part c, the largest oxidation step goes from 25 °C (*ii*) to 25 °C (*iii*), whereas in part d it goes from 200 to 350 °C.

The normalized total intensity of various peaks as a function of temperature is shown in Figure 1c. The data for this plot were generated by fitting all the Ce 4d core-level spectra, similar to those shown in Figures 1a and 1b. The plot is divided into four regions representing different experimental stages: (*i*) in the presence of 0.01 Torr O_2_ which oxidizes the sample, (*ii*) subsequent heating in vacuum which reduces the sample, (*iii*) subsequent dosing of 0.1 Torr H_2_ on the reduced sample, and finally reoxidizing the sample in O_2_ followed by (*iv*) dosing of 0.1 Torr H_2_ on the oxidized sample.

In the first stage (region *i* in Figure 1c) the sample is oxidized by heating in the presence of 0.01 Torr O_2_. Interestingly, the normalized intensities of the features depend on *E*_*h*υ_, indicating that the surface oxidizes less efficiently than the subsurface during annealing in O_2_. A recent theoretical study discusses the preference of the formation of the V_O_ sites on the surface and in the subsurface, which is both facet dependent and V_O_-concentration dependent.^15^ Since a polycrystalline sample is rich in low-coordinated surface sites, less densely packed (110) facets should represent it better than the (111) facets, and indeed V_O_ is more stable on the surface than in the subsurface for the loosely packed facets.^15^ The reason the surface appears not as oxidized as the subsurface can be explained by the thermal reduction counteracting oxidation, which we discuss further in the next paragraph. The oxidation state of the sample remains relatively unchanged as it is cooled down to 25 °C in O_2_.

Once the O_2_ is removed, there is an ever so slight decrease in the normalized intensity of the red features measured at *E*_*h*υ_ = 380 eV and no noticeable change at *E*_*h*υ_ = 780 eV. This indicates a slight reduction to CeO_2-x_ of the surface at 25 °C in vacuum. In the second stage (region *ii* in Figure 1c), the sample is reduced by heating it in vacuum. As the sample temperature is increased to 200 °C in vacuum, we observe an increase in Ce^3+^ intensity due to reduction both on the surface and in the subsurface. A beam-induced reduction can be excluded as we closed the shutter of the beam during heating prior to reaching the target temperature. A potential explanation for this reduction is the reaction of ceria with the H_2_ impurities in the background gas mixture, i.e., the reaction shown in eq 3 (maybe combined with the reaction shown in eq 4) taking place. Indeed, H_2_ can reduce CeO_2_(111) and CeO_2_(100) at high temperatures as shown in refs.^1,16^ Since our polycrystalline thin films are richer in low-coordinated sites than CeO_2_(111) and CeO_2_(100) used in these studies, a lower temperature can already be sufficient to achieve this. However, this cannot be the reason, as we discuss below, that the reduction of the surface and the subsurface is less effective in the presence of H_2_ gas, compared to reduction in vacuum. Instead, we attribute this behavior to thermal reduction. According to ref (17), the formation energy for V_O_ is over 2 eV in Ce_2_O(111) with preference of subsurface V_O_ creation over surface V_O._ However, polycrystalline thin films are full of defect sites which should make thermal reduction more probable compared to single crystals. Indeed, theoretical calculations show that both low-coordination and size effects favor vacancy formation on surfaces compared to fully coordinated bulk regions.^18^ According to a previous experimental study, thermal reduction at 200 °C was observed for two monolayer thick films grown on Pt(111), whereas the ten monolayer thick film started to thermally reduce at 350 °C.^19^ In our experiments, both the surface and the subsurface continue to reduce significantly as the sample temperature is increased further to 350–450 °C (Figure 1c). The thermal reduction of subsurface layers can be explained by the diffusion of V_O_ from the surface to the subsurface layers.

In the third stage (region *iii* in Figure 1c), 0.1 Torr H_2_ is dosed into the chamber, resulting in increased oxidation of both the sample surface and the subsurface at 25 °C. This is in accordance with the proposed homolytic hydride formation mechanism (eq 1).^1,2^ The extent of oxidation remains relatively constant between 25–350 °C. However, as the sample temperature is raised above 350 °C, both the surface and the subsurface appear less oxidized due to surface hydroxyl formation and their recombinative desorption as H_2_O vapor (eqs 3 and 4), as well as the diffusion of hydrides into the bulk.^2^

Contaminant adsorption on the sample surface and competition with reactants are major concerns in surface science experiments at ambient pressures. In these experiments, the main concern is the adsorption of H_2_O impurities, which can cause oxidation of ceria through H_2_O(g) + 3O^2–^ + 2Ce^3+^ + V_O_ → 2O^2–^ + 2OH^–^ + 2Ce^3+^ → H_2_(*g*) + 4O^2–^ + 2Ce^4+^ (reverse reactions of eqs 4 and 3). Interaction of ceria and H_2_O vapor was studied in the literature. TGA and TDS experiments both showed an effective oxidation of the CeO_2-x_ by H_2_O via the recombination of hydroxyls to form H_2_.^20−24^ The competition between this process and the reversible H_2_O desorption (via the recombination of hydroxyls to form H_2_O) was also found to depend on the morphology and crystal orientation.^23,24^ However, the authors of ref (25) find that, contrary to these results, the interaction of H_2_O with partially oxidized ceria does not reoxidize the ceria. These same authors then later claim that strongly reduced ceria does indeed undergo such an oxidation.^26^

The effect of H_2_O vapor was tested by dosing 1 × 10^–6^ Torr H_2_O on a freshly reduced sample. As Figure 1d illustrates, even such a small amount of H_2_O indeed causes oxidation. However, compared to the experiments with H_2_ gas (Figure 1c-*iii*), the temperature-dependent behavior is opposite with H_2_O vapor (Figure 1d), i.e., more oxidation is observed as temperature is increased to 350 °C. This temperature-dependent behavior of H_2_O induced oxidation can be explained by a slow backreaction from surface OH groups to H_2_^17^ (eq 3). This behavior is only reversed at 450 °C, likely due to thermal reduction becoming effective. Another difference between H_2_-induced oxidation through hydride mechanism and H_2_O induced oxidation is that the latter is more pronounced in the subsurface. Due to the distinct temperature-dependent oxidation behavior in H_2_ and H_2_O, we conclude that the underlying chemistry must be different. We suggest that oxidation in the presence of H_2_ is mainly due to hydride formation but H_2_O impurities could also contribute to it.

In the final set of core-level XPS measurements, a freshly prepared oxidized surface was analyzed. As shown in stage *iv* of Figure 1c, the introduction of H_2_ on this sample resulted in the reduction of both the surface and the subsurface as the temperature increased. A comparison with the experiments conducted in vacuum (Figure 1c-*ii*) revealed that the presence of H_2_ led to a lesser degree of reduction in both the surface and the subsurface between 25 and 200 °C. This difference in reduction is interpreted as the interplay between hydride formation and thermal reduction. However, above 350 °C, the initial chemical state of ceria did not significantly impact the final chemical state of the ceria surface (compare Figure 1c stages *iii* and *iv*). In the subsurface, a slight difference remained, with the initially oxidized surface being more oxidized than the initially reduced ceria. At such elevated temperatures, H_2_ constantly interacts with the surface to form hydroxyls, which then recombine to form H_2_O molecules that desorb to the gas phase and leave behind V_O_ sites that are partially filled with hydrides. These sites also diffuse into the subsurface. In short, both at *iii* and *iv* stages in Figure 1c when temperature is increased above 350 °C, the equilibrium chemical state should involve both V_O_ formation (reduction, both thermally and through recombinative H_2_O desorption) and hydride formation (oxidation).

Valence-band spectra were collected in order to corroborate core-level spectra. Figures 2a and 2b show the valence band spectra obtained on reduced and oxidized ceria during the H_2_ dosing experiments. The difference between the spectra obtained in the presence of H_2_ and in vacuum prior to pressurizing the chamber with H_2_ is also displayed. The features that are between 15 and 30 eV are the O 2s and cerium states that are localized core-level electrons which overlap in the energy scale, whereas the features that are between the Fermi level and 11 eV are the hybridized states of O 2p with the electronic states of cerium. More details about the valence band structure of ceria can be found in refs (27 and 28). A noteworthy feature of our spectra is the peak at around 1.2–1.8 eV, which is due to unhybridized Ce 4f states of the Ce^3+^ ions, serving as the fingerprint of reduced ceria.^25^ This assignment was confirmed with resonant photoemission measurements using photon energies between 520–540 eV (not shown): Enhancement was observed for all the valence band features in the 0–11 eV range except for this peak.

**Figure 2:**
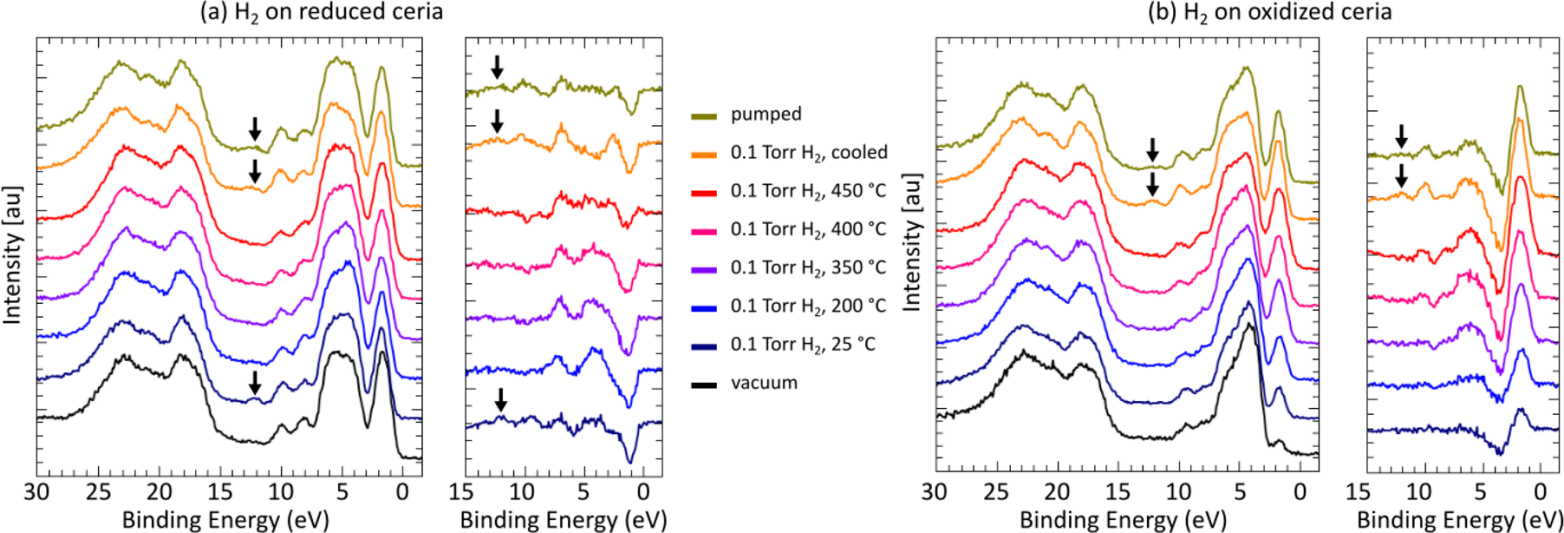
Valence-band spectra with *E*_*h*υ_ = 380 eV of (a) initially reduced ceria and (b) initially oxidized ceria in the presence of 0.1 Torr H_2_. Changes in the spectra in the 0–15 eV range with respect to the spectrum obtained in vacuum are also shown. The peak at the lowest binding energy (around 1.2–1.8 eV) is a fingerprint of Ce^3+^ ions. Arrows indicate a feature that we attribute to molecular H_2_O adsorption.

Figure 2a demonstrates a decrease in the intensity of the peak at 1.2–1.8 eV once H_2_ is dosed, resulting from the oxidation of reduced ceria via hydride formation. The exact position depends on the photon energy, as we do not have the Fermi level to calibrate the photon energy accurately. This decrease in intensity is accompanied by an increase in the intensity of the feature that is around 2.5 eV higher energy than the Ce 4f peak. Similar to the core-level spectra, the oxidation appears more significant in the 25–350 °C range. Above this range, thermal reduction and H_2_O desorption induced reduction processes counteract the oxidation through hydride formation.

The valence band spectra in Figure 2a also exhibit a small feature roughly at 12.5 eV. This peak is apparent only at spectra acquired at 25 °C in the presence of H_2_ or right after H_2_ is removed. We attribute this peak to the 1b_2_ state of molecular H_2_O,^29^ which is bound to surface hydroxyls via H-bonding. 1b_1_ and 3a_1_ states of molecular H_2_O overlap with the ceria states. Resonant photoemission experiments confirm that this peak at 12.5 eV originates from oxygen-containing species and not carbon-containing species due to hydrocarbon contaminants (not shown).

Figure 2b displays the opposite behavior of Figure 2a due to the reduction of oxidized ceria through the formation of hydroxyls, preceding recombinative H_2_O formation and desorption: As H_2_ is dosed and sample temperature is increased, the Ce 4f feature increases in intensity, while the feature approximately 2.5 eV above it decreases in intensity. The adsorbed molecular H_2_O feature at around 12.5 eV is again present but only on the reduced sample. This suggests that H_2_O does not form strong bonds with surface hydroxyls on the oxidized sample. These observations are consistent with the H_2_O peaks observed in the O 1s spectra shown in Section S4. It is worth noting that reduced ceria appears more hydroxylated than oxidized ceria at 25 °C, which explains the facile H_2_O adsorption through H-bonding.

The relative intensity of the 1.2–1.8 eV peak is not the same in the 350–450 °C range for the initially reduced and initially oxidized samples, suggesting a larger involvement of the subsurface, as in Figure 1c.

Figure S9 presents the valence band spectra obtained during thermal treatment in vacuum that results in reduction of oxidized ceria, as well as test experiments with 1 × 10^–6^ Torr H_2_O vapor that results in oxidation of reduced ceria. The changes in the valence band spectra align with the changes observed in the Ce 4d core-levels. The peak that is roughly at 12.5 eV also appears when 1 × 10^–6^ Torr H_2_O vapor is dosed at 25 °C, confirming its association with molecular H_2_O. However, the intensity of this peak is relatively small compared to its intensity in the presence of 0.1 Torr H_2_, which suggests that the partial pressure of H_2_O vapor impurities in 0.1 Torr H_2_ is likely higher than 1 × 10^–6^ Torr.

In summary, using AP-XPS we recorded the changing oxidation state of the initially oxidized and reduced 25 nm thick polycrystalline ceria thin films in the presence of 0.1 Torr H_2_ between 25 and 450 °C. Our findings reveal a complex process of H_2_-splitting on ceria, involving various mechanisms including thermal reduction, reduction through hydroxyl formation followed by recombinative desorption as H_2_O vapor, oxidation through hydride formation, and oxidation through the adsorption of H_2_O impurities. The oxidation state of ceria is determined by the interplay of these processes, shedding light on the intricate nature of the H_2_ interaction with ceria surfaces.

## Supporting Information Available

All XPS spectra, analysis of the O 1s region, and electron microscopy image of the sample (PDF)
Transparent Peer Review report available (PDF)
