# Supplementary material for: Oxidation and Reduction of Polycrystalline Cerium Oxide Thin Films in Hydrogen
Source: J Phys Chem Lett. 2023 Aug 10;14(33):7354–60. doi: 10.1021/acs.jpclett.3c01662 (PMC10461297; doi:10.1021/acs.jpclett.3c01662)
Supplement: Supplementary file 1 — jz3c01662_si_001.pdf [file jz3c01662_si_001.pdf]

Supplementary Information for 'Oxidation and Reduction of Polycrystalline Cerium Oxide Thin Films in Hydrogen'

Adva Ben Yaacov,[1] Lorenz J. Falling[2,3], Roey Ben David,[1] Smadar Attia,[4] Miguel A. Andrés,[1,] Slavomír Nemšák[2,5], Baran Eren,[1,]*

[1]*Department of Chemical and Biological Physics, Weizmann Institute of Science, 234 Herzl Street, 76100 Rehovot, Israel,*

[2]*Advanced Light Source, Lawrence Berkeley National Laboratory, Berkeley, CA 94720, United States of America,*

[3]*Materials Science Division, Lawrence Berkeley National Laboratory, Berkeley, CA 94720, United States of America,*

[4]*Nuclear Research Centre—Negev, Beer-Sheva 84190, Israel,*

[5]*Department of Physics and Astronomy, University of California, Davis, CA 95616, United States of America*

Legend to sections S1-S4

(a-i) pristine, vacuum, 25 °C
(a-ii) vacuum, 200 °C
(a-iii) 0.01 Torr O_2, 200 °C
(a-iv) 0.01 Torr O_2, 25 °C

(b-i) oxidized, vacuum, 25 °C
(b-ii) vacuum, 200 °C
(b-iii) vacuum, 350 °C
(b-iv) vacuum, 400 °C
(b-v) vacuum, 450 °C
(b-vi) reduced, vacuum, 25 °C

(c-i) reduced, 0.1 Torr H_2, 25 °C
(c-ii) 0.1 Torr H_2, 200 °C
(c-iii) 0.1 Torr H_2, 350 °C
(c-iv) 0.1 Torr H_2, 400 °C
(c-v) 0.1 Torr H_2, 450 °C
(c-vi)* 0.1 Torr H_2, 25 °C
(c-vii)* pumped, 25 °C

(d-0)* re-oxidized, vacuum, 25 °C
(d-i) re-oxidized, 0.1 Torr H_2, 25 °C
(d-ii) 0.1 Torr H_2, 200 °C
(d-iii) 0.1 Torr H_2, 350 °C
(d-iv) 0.1 Torr H_2, 400 °C
(d-v) 0.1 Torr H_2, 450 °C
(d-vi) 0.1 Torr H_2, 25 °C
(d-vii)* pumped, 25 °C

(e-i) reduced, vacuum, 25 °C
(e-ii) 10^{-6} Torr H_2O, 25 °C
(e-iii) 10^{-6} Torr H_2O, 200 °C
(e-iv)** 10^{-6} Torr H_2O, 350 °C
(e-v)** 10^{-6} Torr H_2O, 450 °C

* Only VB was measured with $E_{h\upsilon}$ = 380 eV
** in (e), (i-iii) and (iv-v) are from different set of measurements.

S1. Raw XPS data at $E_{h\upsilon}$ = 380 eV

S1.1 Ce 4d

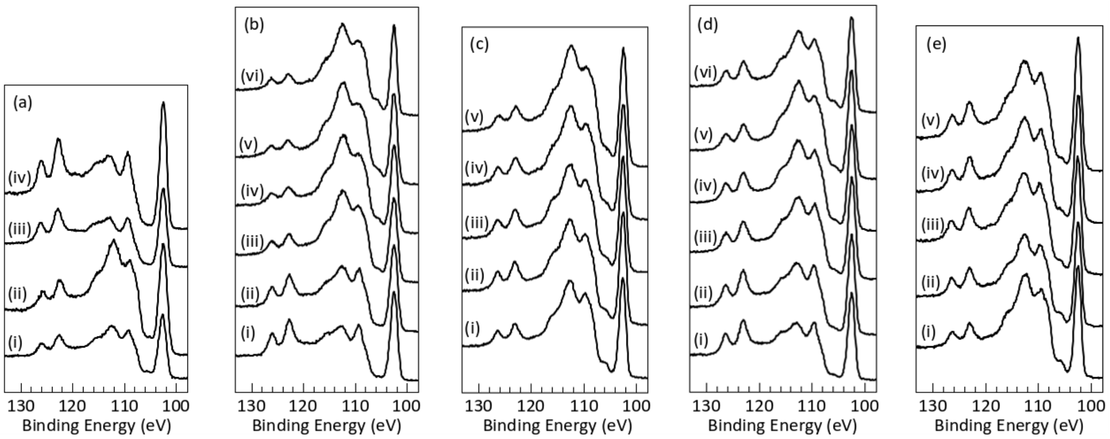

Figure S1 All the Ce 4d spectra obtained during our experiments with $E_{h\upsilon}$ = 380 eV. Energy calibration is performed by setting the Si 2p peak position to 102.5 eV to correct for the inaccuracies in the photon energy.

S1.2 VB

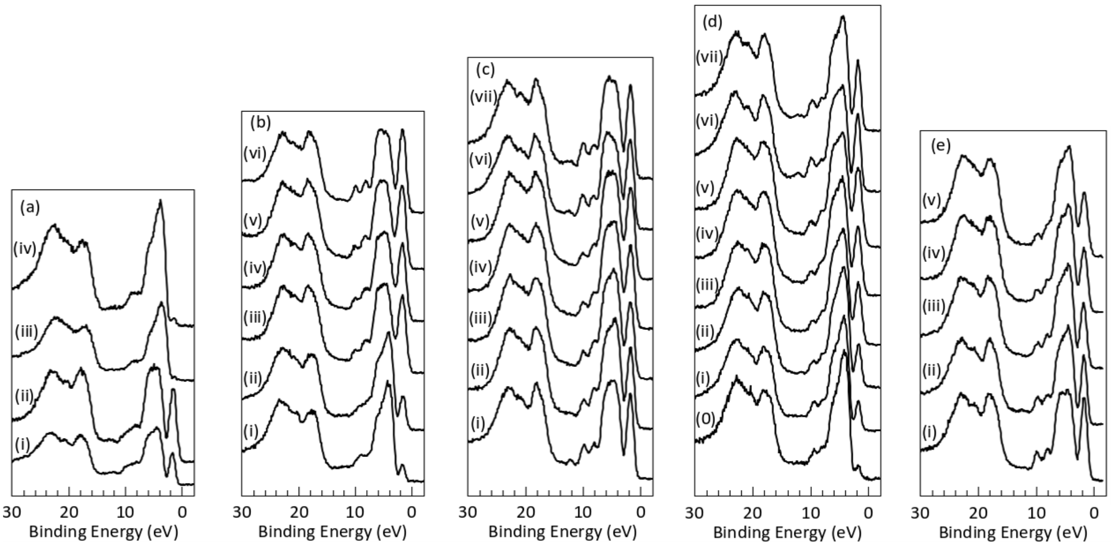

Figure S2 All the valence band spectra obtained during our experiments with $E_{h\upsilon}$ = 380 eV. Since there is no 'known' peak position that could be used as reference, a constant shift to the binding energy was applied to correct for the inaccuracies in the photon energy.

S2. Raw XPS data at $E_{h\upsilon}$ = 530 eV

Although we also acquired valence band spectra with $E_{h\upsilon}$ = 530 eV, this energy corresponds to the absorption energy of the O K absorption edge. As a result, we have some peaks in the valence band that are resonantly enhanced. Therefore, we do not present these spectra here.

Figure S3 reveals the presence of hydrocarbon and oxygenated hydrocarbon contaminants on the surface, characterized by peaks at approximately 284.5 eV and 288.5 eV, respectively. Upon annealing in oxygen, the intensity of the hydrocarbon peak is significantly reduced but not completely eliminated (Figure S3a). While the amount of contaminants varies depending on the conditions, they are not expected to have a significant impact on the normalized intensities of the Ce 4d peaks. Furthermore, these contaminants do not appear to have a major effect on the valence-level spectra, as no resonantly enhanced peaks were observed when measuring the valence band using $E_{h\upsilon}$ = 280-300 eV, near the C K absorption edge. However, it is likely that they contribute to the intensity of the 'hydroxyl' peak in the O 1s region.

S2.1 C 1s

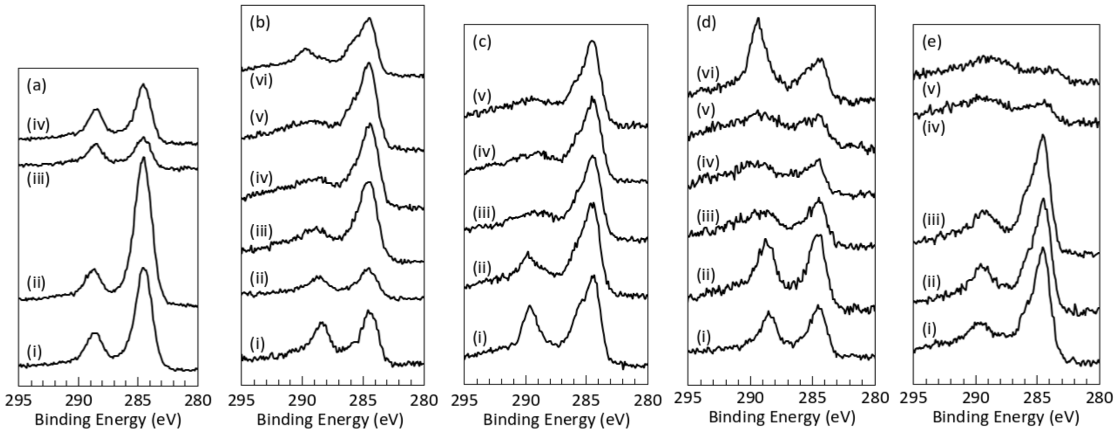

Figure S3 All the C 1s spectra obtained during our experiments with $E_{h\upsilon}$ = 530 eV. Energy calibration is performed by setting the hydrocarbon contamination peak position to 284.5 eV to correct for the inaccuracies in the photon energy.

S2.2 Ce 4d

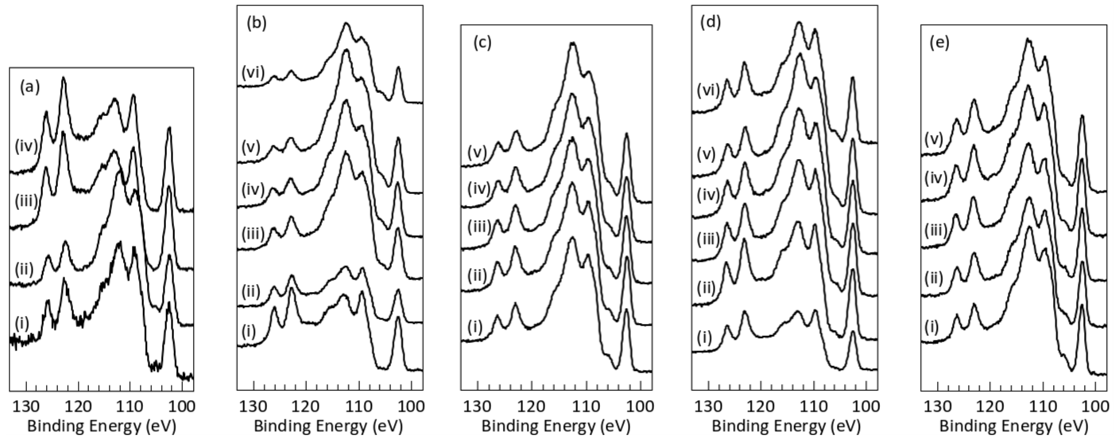

Figure S4 All the Ce 4d spectra obtained during our experiments with $E_{h\upsilon}$ = 530 eV. Energy calibration is performed by setting the Si 2p peak position to 102.5 eV to correct for inaccuracies in the photon energy.

S3. Raw XPS data at $E_{h\upsilon}$ = 780 eV

S3.1 O 1s

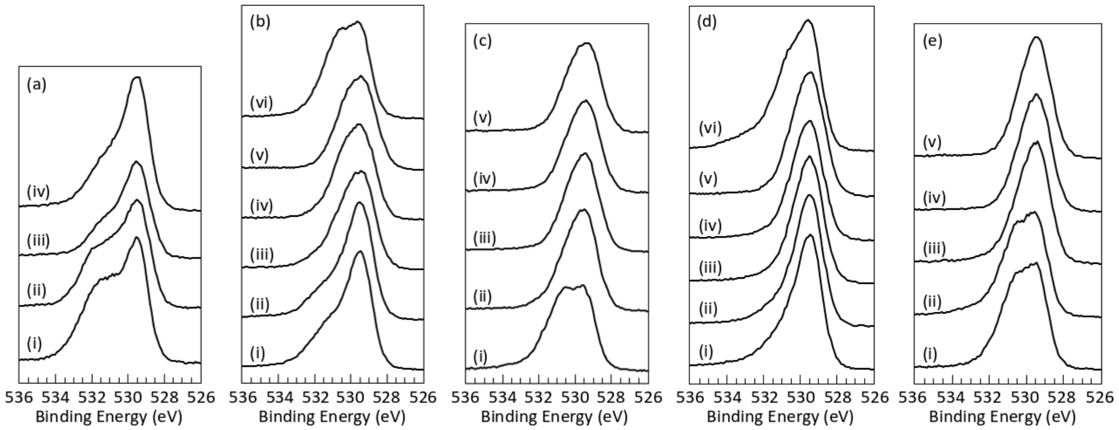

Figure S5 All the O 1s spectra obtained during our experiments with $E_{h\upsilon}$ = 780 eV. Energy calibration is performed by setting the ceria peak position to 529.4 eV to correct for inaccuracies in the photon energy. This is a slightly inaccurate approach, as the peak position for reduced ceria should be higher than that of oxidized ceria. We discuss this more in Section S4 below.

3.2 Ce 4d

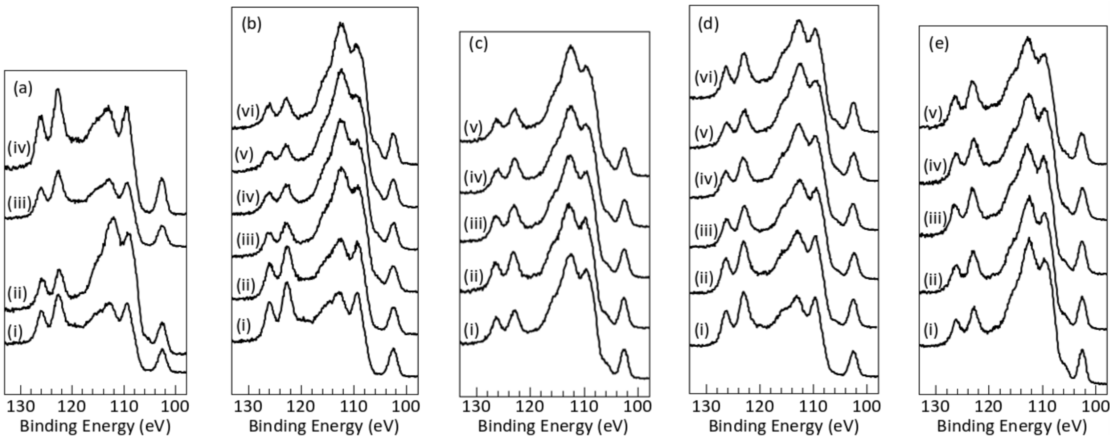

Figure S6 All the Ce 4d spectra obtained during our experiments with $E_{h\upsilon}$ = 780 eV. Energy calibration is performed by setting the Si 2p peak position to 102.5 eV to correct for inaccuracies in the photon energy.

S3.3 VB

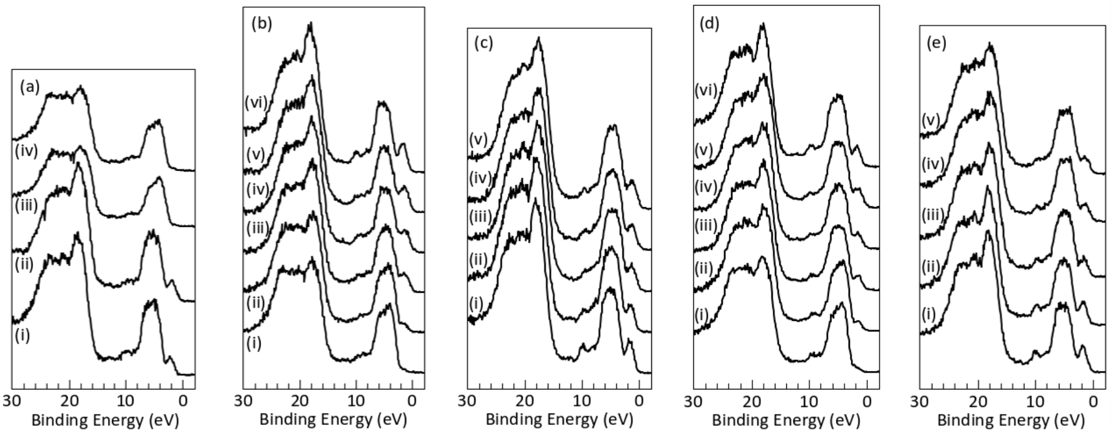

Figure S7 All the valence band spectra obtained during our experiments with $E_{h\upsilon}$ = 780 eV. Since there is no 'known' peak position that could be used as reference, a constant shift to the binding energy was applied to correct for the inaccuracies in the photon energy.

S4. O 1s spectra with fits

Figure S8a is omitted because it is the initial preparation of the sample and is not part of the discussion.

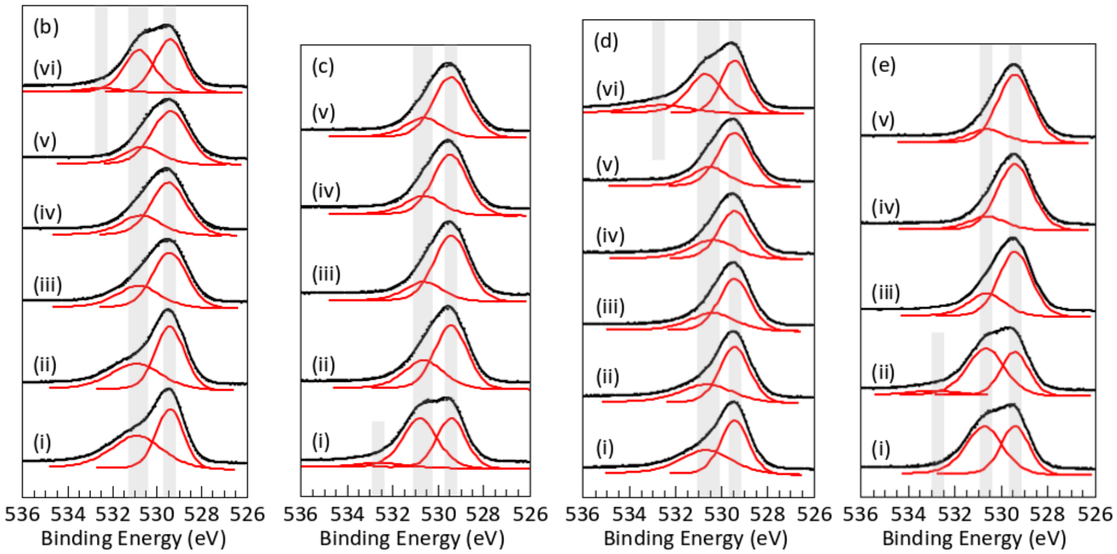

Figure S8 O 1s spectra with fitting.

According to ref. [S1], stoichiometric ceria is expected to exhibit a peak at 530.4 eV. Reduced ceria should have a peak approximately 0.3 eV higher than this peak, while the hydroxyls on reduced ceria should produce a peak around 2.1 eV above the stoichiometric peak.

Ref. [S2] reports that reduced ceria produces a peak at 529.8 eV, and the hydroxyls on reduced ceria exhibit a peak approximately 2 eV higher in energy.

In ref. [S3], stoichiometric and reduced ceria (via ion irradiation) were found to have peaks at 529.4 eV and 530.2 eV, respectively. The hydroxyl peak was observed at 531.7 eV.

Ref. [S4] indicates that stoichiometric ceria generates a peak at 529.4 eV. Reduced ceria exhibits a peak in the range of 529.8-530.0 eV, while hydroxyl groups on reduced ceria produce peaks between 532.0-532.5 eV.

According to ref. [S5], stoichiometric ceria displays a peak at 529.5 eV. The adsorption of H_2O on stoichiometric ceria leads to the emergence of the hydroxyl peak at 531.5 eV and a molecular H_2O peak above 533.5 eV. In the same study, the reduced ceria peak was found to be approximately 530 eV, with the hydroxyl peak shifting to around 532 eV upon H_2O adsorption, and the molecular H_2O peak appearing above 533.5 eV.

Ref. [S6] suggests that oxygen anions adjacent to Ce^{4+} cations have a peak with a binding energy of 529.3 eV for CeO_2 and 529.7 eV for CeO_{2-x}. It is claimed that oxygen anions next to Ce^{3+} cations in CeO_{2-x} exhibit a peak with a binding energy of 532.0 eV. The OH/H_2O peak was reported to appear at 530.9 eV for CeO_2 and 531.7-532.0 eV for CeO_{2-x}.

In our study, the main peak is fixed at 529.4 eV, which is an oversimplification because the binding energy should increase as the surface becomes more reduced. Consequently, the hydroxyl peaks appear at relatively lower energies when the surface is reduced. Due to variations in photon energy and the absence of a Fermi level in ceria, it is challenging to precisely adjust the binding energies in each experiment.

S5. Additional valence band spectra

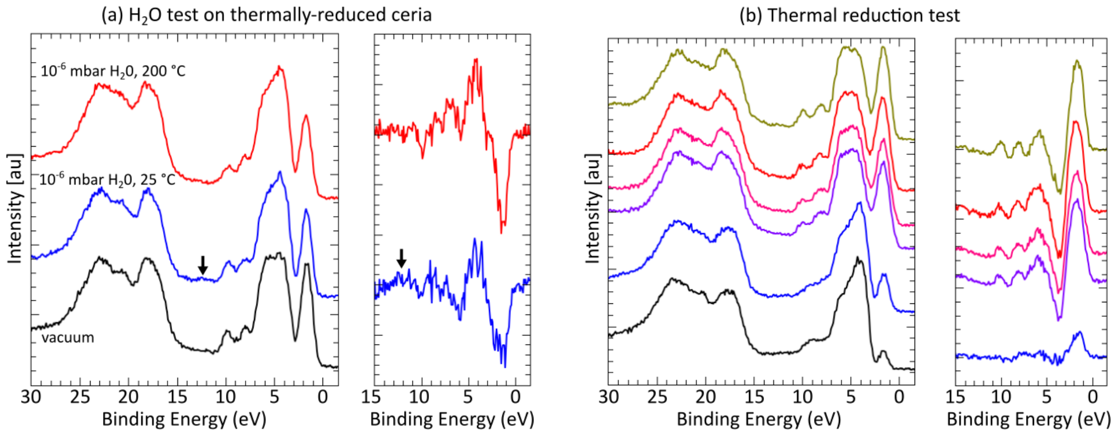

Figure S9 Valence band spectra and their difference with respect to the spectra obtained in vacuum: (a) in the presence of 10^{-6} Torr H_2O vapor, (b) during heating in vacuum. (b) has the same color code as in Figure 2. The arrows in (a) point a small peak due to adsorbed molecular H_2O on the surface.

S6. Discussion on O 1s spectra

The features observed in the O 1s region in this study exhibit broadness due to two main factors. Firstly, the monochromatization of the X-ray beam at beamline 9.3.2 is less effective at $E_{h\upsilon}$=780 eV compared to lower photon energies. Secondly, the sample consists of two oxidation states, resulting in broadening of the oxide features. The broadness of the hydroxyl features can also be attributed to the various adsorption sites of hydroxyls on the surface and grain boundaries. Consequently, quantitative analysis is not feasible, but several qualitative deductions can be made.

Before fitting the O 1s region spectra (Figure S8), an energy calibration was performed by shifting the spectrum so that the binding energy of the oxide peak was fixed at 529.4 eV. The spectra (Figure S8) exhibited additional features, particularly those located 1.2-1.5 eV higher than the oxide peak. Here is a summary of the significant outcomes from the O 1s data:

- The broad peaks observed at ~530.6-530.9 eV are attributed to hydroxyls, the native oxide of the Si substrate, and oxygenated hydrocarbons. The intensity of this peak is lowest in the 350-450 °C temperature range, likely due to the absence of hydroxyls. At 25 °C, regardless of the gas mixture, the intensity of this peak is highest, indicating surface hydroxylation.

- On oxidized surfaces, the 'hydroxyl peak' appears broader at 25 °C, whereas on reduced surfaces, it appears narrower. This difference may be related to the types of hydroxyls present (terminal vs bridging). However, the spectral resolution in the O 1s region is not sufficient to provide further insights into this distinction. Additionally, this peak comprises additional components.

-Reduced ceria is significantly more hydroxylated than oxidized ceria at 25 °C.

- Even under HV conditions, the surfaces are covered with hydroxyls at 25 °C due to the presence of background gases such as H_2 and H_2O. On reduced surfaces at 25 °C, a small amount of adsorbed molecular H_2O is typically observed in the energy range of 532-532.5 eV.

S7. Scanning electron microscopy image of the sample

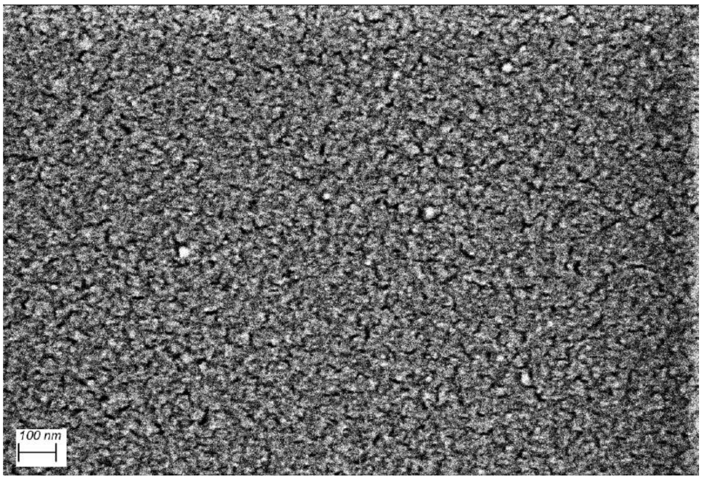

Figure S10 Scanning electron microscopy (SEM) image of an as-prepared sample used in XPS studies. Image was acquired with a Gemini SEM 500 from Zeiss. We used 2 kV acceleration energy and secondary electron detection.

References

[S1] Mullins, D. R; Overbury, S. H.; Huntley, D. R. Electron Spectroscopy of Single Crystal and Polycrystalline Cerium Oxide Surfaces. Surf. Sci. 1998, 409, 307–319.

[S2] Li, Z. R.; Werner, K.; Qian, K.; You, R.; Plucienik, A.; Jia, A.; Wu, L. H.; Zhang, L. Y.; Pan, H. B.; Kuhlenbeck, H.; Shaikhutdinov, S.; Huang, W. X.; Freund, H. J. Oxidation of Reduced Ceria by Incorporation of Hydrogen. Angew. Chem., Int. Ed. 2019, 58, 14686–14693.

[S3] Maslakov, K. I.; Teterin, Y. A.; Popel, A. J.; Teterin, A. Y.; Ivanov, K. E.; Kalmykov, S. N.; Petrov, V. G.; Petrov, P. K.; Farnan, I. XPS Study of Ion Irradiated and Unirradiated CeO2 Bulk and Thin Film Samples. Appl. Surf. Sci. 2018, 448, 154–162.

[S4] Li, Z. R.; Werner, K.; Chen, L.; Jia, A. P.; Qian, K.; Zhong, J. Q.; You, R.; Wu, L. H.; Zhang, L. Y.; Pan, H. B.; Wu, X. P.; Gong, X. Q.; Shaikhutdinov, S.; Huang, W. X.; Freund, H. J. Interaction of Hydrogen with Ceria: Hydroxylation, Reduction, and Hydride Formation on the Surface and in the Bulk. Chem.─Eur. J. 2021, 27, 5268–5276.

[S5] Matolín, V.; Matolínová, I.; Dvořák, F.; Johánek, V.; Mysliveček, J.; Prince, K.C.; Skála, T.; Stetsovych, O.; Tsud, N.; Václavů, M.; Šmíd, B. Water Interaction with CeO_2(1 1 1)/Cu(1 1 1) Model Catalyst Surface. Catalysis Today 2012, 181, 124–132.

[S6] Lykhach, Y.; Johánek, V.; Aleksandrov, H. A.; Kozlov, S. M.; Happel, M.; Skála, T.; Petkov, P. S.; Tsud, N.; Vayssilov, G. N.; Prince, K. C.; Neyman, K. M.; Matolín, V.; Libuda, J. Water Chemistry on Model Ceria and Pt/Ceria Catalysts. J. Phys. Chem. C 2012, 116, 12103−12113.
