# Supplementary material for: Oxidation and Reduction of Polycrystalline Cerium Oxide Thin Films in Hydrogen
Source: J Phys Chem Lett. 2023 Aug 10;14(33):7354–60. doi: 10.1021/acs.jpclett.3c01662 (PMC10461297; doi:10.1021/acs.jpclett.3c01662)
Supplement: Supplementary file 2 — jz3c01662_si_002.pdf [file jz3c01662_si_002.pdf]

jz-2023-01662r.R1

Name: Peer Review Information for "Oxidation and Reduction of Polycrystalline Cerium Oxide Thin Films in Hydrogen"

First Round of Reviewer Comments

Reviewer: 1

Comments to the Author

This study deals with the interaction of H_2 with ceria surfaces using hydrogen to investigate the surface and subsurface oxidation state of ceria thin films under operando conditions in the temperature range of 25-450°C using ambient pressure X-ray photoelectron spectroscopy. The authors' results show that the oxidation state during hydrogen interaction involves a complex interplay between oxidising hydride formation, reducing thermal reduction and reducing formation of hydroxyls followed by water desorption.

Although this is a relatively systematic study, one big question is the presence of silicon oxide in the Ce4d spectra measured from a 25 nm thick layer of ceria deposited on a silicon substrate, giving the impression of significant contamination of the layers studied. How do the authors explain this phenomenon when the photon energies used give an inelastic mean free path (IMFP) of electrons in CeO_2 of about 0.7 nm and 1.3 nm respectively?

Why didn't the authors use another inert substrate, or didn't they passivate the silicon sufficiently?

In Figure 3 it is not clear at what photon energy the VB spectra were recorded, it can only be inferred. But why did the authors not use photons of such energies for VB that the depth of information was the same as for the aforementioned core-level spectra, whose behaviour they are supposed to confirm?

There are a few typos, e.g:

Supplementary information, S4. O 1s spectra with fits, penultimate paragraph: The OH/H_2O peak is reported to occur at 590.9 eV for CeO_2, which is obviously an incorrect value.

Reviewer: 2

Comments to the Author

The article “Oxidation and Reduction of Polycrystalline Cerium Oxide Thin Films in Hydrogen” provides insight into the complex and temperature-dependent behavior of ceria in hydrogen atmosphere using detailed in-situ XPS analysis. The authors showed that depending on temperature and initial oxidation state, hydrogen can lead to either reduction or oxidation of ceria. The results complement well with existing ex-situ studies.

Some minor changes are suggested:

Material

It would be good if the cerium oxide layer and its production could be described in a more detail to allow for repeatability.

Experiment

What was the heating rate and how long was the holding time of the steps in Fig. c? Did you measure immediately after the temperature steps were reached or did you wait? Do you think the sample was in equilibrium when you measured, or is it possible that oxidation states change when held at a certain temperature?

Fig. 2

Perhaps place the "H2 on reduced" also side by side next to the "H2O on reduced" for better comparison. Maybe point out directly in the diagram that for H2 the largest oxidation step goes from 25 (ii) to 25 (iii) and for H2O from 200(iii) to 350(iii).

Si2p peak

You calculated an IMFP of 0.7 nm and 1.3 nm and state that your ceria film on top of the SI wafer is 25 nm thick (~20*IMFP). Thereby, it should be impossible to see any Si. Yet, you see quite a big Si2p peak in your spectra. An explanation for this is needed. Is your ceria film non continuous?

H2O

I am not entirely convinced yet that H2O can be ruled out as the cause of partial oxidation of reduced CeO2-x. As I understood, for H2 most oxidation seems to occur already at 25 °C and then remains relatively constant, whereas for H2O oxidation increases with temperature. However, you have shown that H2O at 1×10-6 causes oxidation, and saw that the amount of H2O in H2 gas is even greater than that. Maybe at higher H2O partial pressure the oxidation at 25 °C is already significant. While at some points the wording could be a little more tentative, I think it's mostly appropriate.

Spelling

P5. l13.: “spit-orbit” -> “spin-orbit”

P11. L6.: “oxidation though hydride” -> “oxidation through hydride”

Author's Response to Peer Review Comments:

Dear Prof. Editor,

We would like to express our gratitude to you for the peer review process. We also would like to thank the reviewers for their comments and suggestions. Below, we provide a point-by-point response to the raised issues. While the comments of the reviewers are shown in *italic* font, our responses are shown in regular font. Highlighted parts are additions or changes in the manuscript.

Reviewer #1:

Recommendation: *This paper is probably publishable, but major revision is needed; I do not need to see future revisions.*

We cordially thank the reviewer for their comments.

Comments:
1a - This study deals with the interaction of H_2 with ceria surfaces using hydrogen to investigate the surface and subsurface oxidation state of ceria thin films under operando conditions in the temperature range of 25-450°C using ambient pressure X-ray photoelectron spectroscopy. The authors' results show that the oxidation state during hydrogen interaction involves a complex interplay between oxidizing hydride formation, reducing thermal reduction and reducing formation of hydroxyls followed by water desorption. Although this is a relatively systematic study, one big question is the presence of silicon oxide in the Ce4d spectra measured from a 25 nm thick layer of ceria deposited on a silicon substrate, giving the impression of significant contamination of the layers studied. How do the authors explain this phenomenon when the photon energies used give an inelastic mean free path (IMFP) of electrons in CeO_2 of about 0.7 nm and 1.3 nm respectively?

There is indeed some Si debris on the samples, which is typical for Si wafers that are cleaved or broken into pieces. However, the density of the Si debris on the surface is 1-2% and cannot explain the intense Si peaks. In our measurements, the normalized intensity of the Si 2p peak is roughly 1/3rd of the total intensity of the Ce 4d peaks after taking the photoionization cross sections into account. The reason behind this is the low packing factor of our ceria films. When a thin film is prepared with an energetic method such a magnetron sputtering, the packing factor is close to unity. However, for samples prepared by thermal evaporation and e-beam evaporation, the packing factor is typically between 70% and 90%. The sample temperature during the growth of our ceria films was room temperature, so we should expect a packing factor close to the lower end of this scale, that is, 70%.

The SEM image below shows an example of our sample with secondary electron detection. The grains appear separated by wide grain boundaries, i.e., low packing factor. This means some of the native oxide covered Si substrate is exposed. Photoelectrons generated in the native oxide in these exposed boundary regions can travel without being inelastically scattered by ceria and reach the electron analyzer. This is the reason behind the observed Si peaks. In fact, their presence is advantageous for this study because their peak positions serve as excellent reference for the Ce 4d region.

We added the following sentence to the page 4 of the main text:

A 25-nm thick polycrystalline ceria thin film prepared by e-beam evaporation on a highly doped silicon wafer piece was used as our sample. The substrate was kept at 25°C during deposition. The deposition rate was 0.2-0.3 Å/s.

We made the following additions to the page 5 of the main text:

The purple peak in Figs. 1a and 1b is due to Si2p peak of the native oxide of the silicon wafer substrate, which remains unaffected by the changing conditions. The normalized intensity of the Si2p peak is around 1/3rd of the normalized total intensity of the Ce4d peaks. Such a high Si2p intensity is because of the relatively low packing factor of the ceria thin films (Fig. S10), and therefore, some of the underlying Si is exposed.

The following image is added to the SI:

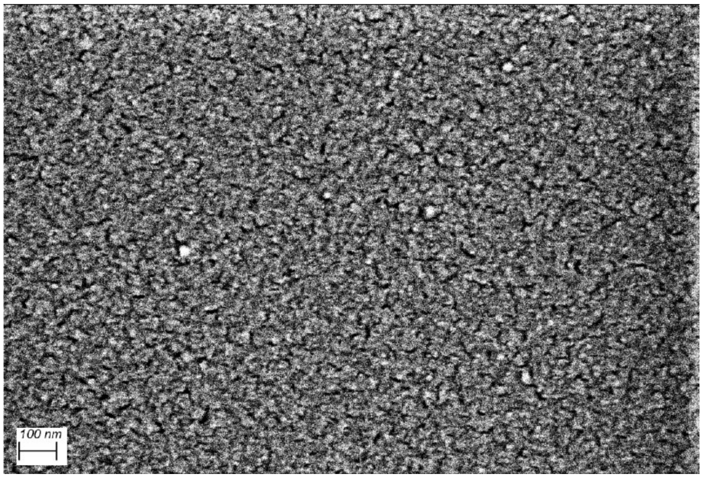

Fig. S10 Scanning electron microscopy (SEM) image of an as-prepared sample used in XPS studies. Image was acquired with a Gemini SEM 500 from Zeiss. We used 2 kV acceleration energy and secondary electron detection.

1b- Why didn't the authors use another inert substrate, or didn't they passivate the silicon sufficiently?

In our experience, Si is the best choice as a substrate for studying thin films or nanoparticles attached to it. This is because the native oxide layer formed on it is chemically inert. There is no need for passivation of the Si surface, as long as the native oxide is intact. Moreover, since Si wafer pieces used in this study are heavily doped, they do not cause any charging related issues.

Other groups prefer using Au foil instead of heavily doped Si. This is also a decent choice, but Au atoms become mobile at high temperatures and can wet the ceria surface. Au-ceria interface might also not be as inactive as desired because of a potential strong metal-support interaction (SMSI).

2- In Figure 3 it is not clear at what photon energy the VB spectra were recorded, it can only be inferred. But why did the authors not use photons of such energies for VB that the depth of information was the same as for the aforementioned core-level spectra, whose behaviour they are supposed to confirm?

VB spectra that are shown in the main text were recorded with 380 eV photon energy. We now indicate this in the figure caption. The photon flux of the beamline decreases significantly as the photon energy is lowered. This is the reason why we do not have VB spectra that are as surface sensitive at the core-level spectra.

We did not say we performed VB spectra to confirm the core-level spectra, rather to corroborate it. We also did not do any temperature-dependent trend analysis with VB spectra, because this would be just replicating what we already present with the core-level spectra. Therefore, we do think that our VB spectra serve their purpose well even with 380 eV photon energy. Moreover, they provide additional information in the form of molecular water adsorption on the surface.

The following is added to the figure caption:

Valence-band spectra with $E_{h\upsilon}$=380 eV of (a) initially reduced ceria and (b) initially oxidized ceria in the presence of 0.1 Torr H_2.

3- There are a few typos, e.g:Supplementary information, S4. O 1s spectra with fits, penultimate paragraph: The OH/H_2O peak is reported to occur at 590.9 eV for CeO_2, which is obviously an incorrect value.

We corrected this mistake. We also did couple of rounds of proofreading and corrected the typos we found.

Additional Questions:

Urgency: Moderate

Significance: High

Novelty: Moderate

Scholarly Presentation: High

Is the paper likely to interest a substantial number of physical chemists, not just specialists working in the authors' area of research?: No

Reviewer #2

Recommendation: *This paper is publishable subject to minor revisions noted. Further review is not needed.*

We cordially thank the reviewer for their comments.

Comments:
The article "Oxidation and Reduction of Polycrystalline Cerium Oxide Thin Films in Hydrogen" provides insight into the complex and temperature-dependent behavior of ceria in hydrogen atmosphere using detailed in-situ XPS analysis. The authors showed that depending on temperature and initial oxidation state, hydrogen can lead to either reduction or oxidation of ceria. The results complement well with existing ex-situ studies.

Some minor changes are suggested:

1- Material

It would be good if the cerium oxide layer and its production could be described in a more detail to allow for repeatability.

Please refer to our answer to first reviewer's question 1a above.

2- Experiment

What was the heating rate and how long was the holding time of the steps in Fig. c? Did you measure immediately after the temperature steps were reached or did you wait? Do you think the sample was in equilibrium when you measured, or is it possible that oxidation states change when held at a certain temperature?

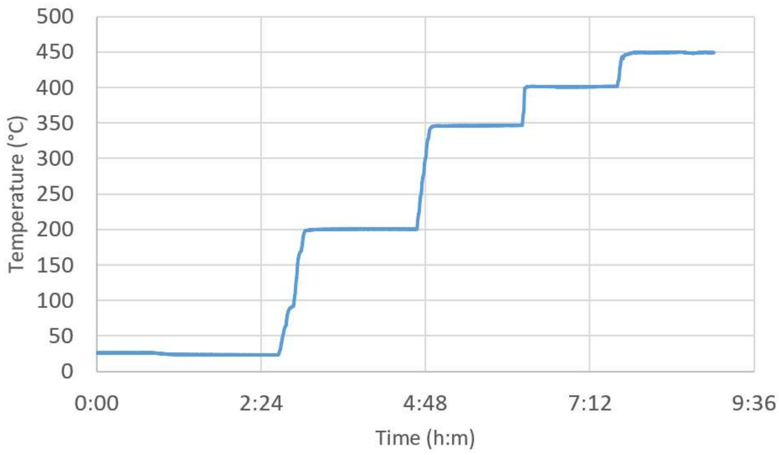

The diagram above shows the heating profile from one set of our experiments. Heating rate is lower at low temperatures (around 7 °C/min), which increases to 10 °C/min, and 15 °C/min as the temperature is increased. Acquisition of a full set of data takes around 1 h and 20 min. In each set, we first start with $E_{h\upsilon}$=780 eV measurements, then continue with $E_{h\upsilon}$=530 eV, and $E_{h\upsilon}$=380 eV measurements. Before the first measurement, we wait for a couple of minutes. In addition, we first acquire a survey spectrum that takes another couple of minutes. So, there is around 5 minutes waiting time prior to each measurement. Especially at elevated temperatures, near-equilibrium (we cannot claim equilibrium without doing a test experiment) is probably reached within 5 minutes, but there might be gradual changes at lower temperatures. In order to compensate for this, we wait longer (roughly 10 more minutes) right after we dose a gas at room temperature. In some of the conditions, we measured the Ce4d region with the same photon energy twice to account for any major changes with time. While we did not observe any major changes in the spectra with time, we cannot fully exclude very gradual changes. We also do not think it is possible to exclude such gradual changes in any experiment of this type, unless it is a dedicated time-dependent study in the course of ~ ten hours. An example of such a study can be found here: ACS catalysis 2022, 12, 13, 7709–7718

3- Fig. 2

Perhaps place the "H_2 on reduced" also side by side next to the "H2O on reduced" for better comparison. Maybe point out directly in the diagram that for H2 the largest oxidation step goes from 25 (ii) to 25 (iii) and for H_2O from 200(iii) to 350(iii).

This is a good idea. We changed Fig. 2 into Fig. 1d. We also added the following to the figure caption:

On reduced samples, in (c) the largest oxidation step goes from 25°C (*ii*) to 25°C (*iii*), whereas in (d) from 200°C to 350°C.

4- Si2p peak

*You calculated an IMFP of 0.7 nm and 1.3 nm and state that your ceria film on top of the SI wafer is 25 nm thick (~20*IMFP). Thereby, it should be impossible to see any Si. Yet, you see quite a big Si2p peak in your spectra. An explanation for this is needed. Is your ceria film non continuous?*

Please refer to our answer to first reviewer's question 1a above.

In addition, we changed 1.3 nm to 1.2 nm. There was a slight error.

5- H_2O

I am not entirely convinced yet that H2O can be ruled out as the cause of partial oxidation of reduced CeO2-x. As I understood, for H2 most oxidation seems to occur already at 25 °C and then remains relatively constant, whereas for H2O oxidation increases with temperature. However, you have shown that H2O at 1×10-6 causes oxidation, and saw that the amount of H2O in H2 gas is even greater than that. Maybe at higher H2O partial pressure the oxidation at 25 °C is already significant. While at some points the wording could be a little more tentative, I think it's mostly appropriate.

Inevitably, there will be some oxidation of reduced ceria caused by H_2O impurities. The question is how much. It is true that the partial pressure of H_2O in 0.1 mbar H_2 is more than 1e-6 Torr, as evidenced by our valence-level spectra. However, oxidation via water is a slow process, taking place at elevated temperatures. This is because of the high energy barrier of recombinative desorption of adsorbed hydroxyls on the surface (see ref. 17). This is why we currently think that hydride formation should be the main reason behind oxidation of reduced ceria at 25 °C.

We agree with the reviewer about the wording. We therefore did the following change:

We suggest that oxidation in the presence of H_2 is mainly due to hydride formation, but H_2O impurities could also contribute to it.

We also did the following change in the abstract:

We find that although 1×10^{-6} Torr water vapor oxidizes ceria, it is probably not the primary driver behind the oxidation of reduced ceria in the presence of hydrogen.

6- Spelling

P5. l13.: "spit-orbit" -> "spin-orbit"

P11. L6.: "oxidation though hydride" -> "oxidation through hydride"

We corrected these mistakes. We also did couple of rounds of proofreading and corrected the typos we found.

Additional Questions:

Urgency: High

Significance: High

Novelty: High

Scholarly Presentation: High

Is the paper likely to interest a substantial number of physical chemists, not just specialists working in the authors' area of research?: Yes
